# Assessing the Dermal Penetration Efficacy of Chemical Compounds with the Ex-Vivo Porcine Ear Model

**DOI:** 10.3390/pharmaceutics14030678

**Published:** 2022-03-19

**Authors:** Cornelia M. Keck, Ayat Abdelkader, Olga Pelikh, Sabrina Wiemann, Vasudha Kaushik, David Specht, Ralph W. Eckert, Reem M. Alnemari, Henriette Dietrich, Jana Brüßler

**Affiliations:** Department of Pharmaceutics and Biopharmaceutics, Philipps-University of Marburg, Robert-Koch-Str. 4, 35037 Marburg, Germany; ayat.abdelkader@pharmazie.uni-marburg.de (A.A.); olga.pelikh@pharmazie.uni-marburg (O.P.); sabrina.wiemann@pharmazie.uni-marburg.de (S.W.); vasudha.kaushik@pharmazie.uni-marburg.de (V.K.); david.specht@pharmazie.uni-marburg.de (D.S.); ralph.w.eckert@googlemail.com (R.W.E.); ph.dreem@hotmail.com (R.M.A.); henriette.dietrich@staff.uni-marburg.de (H.D.); jana_bruessler@web.de (J.B.)

**Keywords:** skin, dermal penetration, nanocarrier, nanocrystals, smartFilms, NLC, lipid nanoparticles

## Abstract

(1) Background: The ex vivo porcine ear model is often used for the determination of the dermal penetration efficacy of chemical compounds. This study investigated the influence of the post-slaughter storage time of porcine ears on the dermal penetration efficacy of chemical compounds. (2) Methods: Six different formulations (curcumin and different fluorescent dyes in different vehicles and/or nanocarriers) were tested on ears that were (i) freshly obtained, (ii) stored for 24 or 48 h at 4 °C after slaughter before use and (iii) freshly frozen and defrosted 12 h before use. (3) Results: Results showed that porcine ears undergo post-mortem changes. The changes can be linked to rigor mortis and all other well-described phenomena that occur with carcasses after slaughter. The post-mortem changes modify the skin properties of the ears and affect the penetration efficacy. The onset of rigor mortis causes a decrease in the water-holding capacity of the ears, which leads to reduced penetration of chemical compounds. The water-holding capacity increases once the rigor is released and results in an increased penetration efficacy for chemical compounds. Despite different absolute penetration values, no differences in the ranking of penetration efficacies between the different formulations were observed between the differently aged ears. (4) Conclusions: All different types of ears can be regarded to be suitable for dermal penetration testing of chemical compounds. The transepidermal water loss (TEWL) and/or skin hydration of the ears were not correlated with the ex vivo penetration efficacy because both an impaired skin barrier and rigor mortis cause elevated skin hydration and TEWL values but an opposite penetration efficacy. Other additional values (for example, pH and/or autofluorescence of the skin) should, therefore, be used to select suitable and non-suitable skin areas for ex vivo penetration testing. Finally, data from this study confirmed that smartFilms and nanostructured lipid carriers (NLC) represent superior formulation strategies for efficient dermal and transdermal delivery of curcumin.

## 1. Introduction

Porcine skin has a high similarity to human skin, and thus, is often used as a model for human skin [1,2,3,4,5]. The ex vivo porcine ear model has various applications; in most cases, it is used for the determination of the dermal penetration efficacy of chemical compounds from different formulations or to determine skin effects of the application of chemical compounds and/or topical formulations [2,6,7,8]. In many cases, porcine skin is obtained from pig ears. From the ears, skin can be removed for further testing, or alternatively, tests can be directly conducted on the intact ear. The latter test model has the beauty that the skin remains connected to the cartilage of the tissue, which enables the maintenance of the skin tension, thus mimicking highly physiological skin conditions. Due to this, the ex vivo model—in comparison to classical penetration models, i.e., diffusion cells—allows for more detailed mechanistic investigations into the dermal penetration of chemical compounds and can test the influence of the formulation on skin properties at the same time [9,10].

Porcine skin and ears can be used in a fresh state or can be frozen and defrosted on demand. Frozen and defrosted skin and ears are most frequently used and recommended for simple penetration testing, whereas fresh skin and ears are recommended when significant biotransformation of the test compound in the skin is expected and/or if dermatopharmacokinetic studies are conducted [8,11]. To date, the term “fresh” is mainly used to clearly state that the skin and ears that were used for the presented skin study were not frozen and defrosted before use. The use of fresh ears can, therefore, mean that freshly obtained ears were directly used after they were collected from the slaughterhouse or at later time points, i.e., after storage for 24 to 72 h [5,6,8,12,13,14,15,16,17,18,19,20,21,22,23,24,25,26,27,28,29,30,31,32,33,34,35,36,37]. A question that remains unanswered so far is if the storage time of the ears after slaughter affects the penetration efficacy of chemical compounds. Hence, to date, a systematic study that investigates the influence of the post-slaughter age of pig ears on the ex vivo dermal penetration efficacy of chemical compounds is not available. This means that at present, it is not known if ears of different post-slaughter ages lead to comparable results or not. However, when conducting skin research, and especially, when frequently assessing the ex vivo dermal penetration efficacy of different formulations, such knowledge is of uppermost importance. The important but non-foreseeable effect of the vehicle on the dermal penetration efficacy of different chemical compounds and the need for individual testing is well-described in the literature [38,39]. Consequently, this study aimed to investigate the influence of post-slaughter ear age on the ex vivo dermal penetration efficacy of different chemical compounds from different vehicles.

## 2. Materials and Methods

### 2.1. Materials

The study was conducted with six different formulations that were developed and thoroughly characterized regarding their physical-chemical properties in previous studies (Table 1). Formulations were selected that contained different fluorescent ingredients but also different classical and innovative formulation principles (liquid, semi-solid and solid formulations). At the same time, the formulations also possessed some similarities, which allowed for a direct comparison of the results.

**Figure 1:**
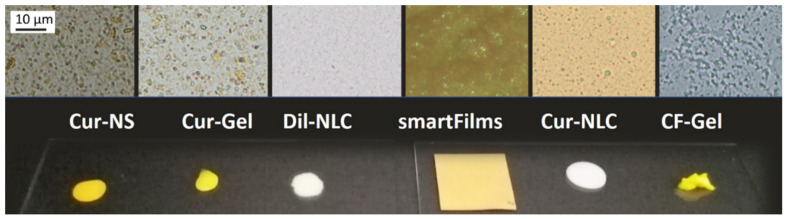
Microscopic images (upper: image sections of 1000-fold magnifications for Cur-NS, Cur-Gel, DiI-NLC, Cur-NLC and CF-Gel, and 50-fold magnification for smartFilms (scale bar corresponds to 5 mm) and macroscopic images (lower) of the formulations used in this study.

Based on these considerations, curcumin was used as the model drug and formulated in four different vehicles, i.e., nanocrystals (drug particles of submicron size in suspension = nanosuspension), nanocrystals in hydrogel, as a dermal patch (smartFilms) and encapsulated in nanostructured lipid carriers (NLCs). In addition, NLCs loaded with the lipophilic dye DiI perchlorate (DiI) and a collagen gel loaded with the fluorescent dye 6-carboxyfluorescein (CF) were used. Table 1 provides an overview of the formulations tested, their compositions and their physical-chemical characteristics. Figure 1 provides macroscopic and microscopic images of the different formulations. Appendix A provides a list of abbreviations.

As it is well-known that changes in physical-chemical properties of the formulations, i.e., changes in the size or crystalline state, can strongly influence the penetration results, it was important to ensure that samples maintained their physical-chemical properties throughout the entire study (three weeks). If samples are not physically stable, changes typically occur quickly after production. Hence, the speed of changes in the physico-chemical properties of the samples decline during storage. Therefore, all formulations were produced >3 months ahead of the study and characterized regarding their physical-chemical properties (particle size (determined by dynamic light scattering, laser diffraction and microscopic analysis), zeta potential (ZP) and crystalline state) throughout the study (Table 1).

### 2.2. Methods

#### 2.2.1. Experimental Setup

The influence of the post-mortem age of porcine ears on the penetration efficacy of chemical compounds was investigated by using four kinds of ears, i.e., fresh ears (A-ears: used within 4–12 h after slaughter), fresh ears stored for 24 h at 4 °C before use (B-ears), fresh ears stored for 48 h at 4 °C before use (C-ears) and frozen and defrosted ears (D-ears: frozen in a fresh state (−20 °C ± 2 °C) and defrosted 12 h before use). The experiment for the fresh ears was performed in triplicate, i.e., on three subsequent weeks in July/August 2020 (trials 1–3). An additional experiment was conducted with A-ears and D-ears in September 2020 (trial 4, Table 2). For the determination of the penetration efficacy, the different formulations (cf. Table 1) were applied on the different ears and the penetration efficacy was determined after 6 h of penetration at 32 °C (Table 2). The skin parameters of untreated skin, i.e., transepidermal water loss (TEWL), skin hydration, skin friction, skin antioxidant capacity (AOC), stratum corneum thickness (SCT) and autofluorescence of the skin (AF), were also determined for each trial and the differently aged ears (Table 2).

#### 2.2.2. Dermal Penetration Testing

Ears were freshly obtained from a local slaughterhouse, carefully washed with lukewarm water (approx. 23–25 °C) and dried with paper towels. Thereafter, they were randomly allocated into three groups. Group-A ears were used immediately, and group-B and -C ears were placed in a plastic box (each group in one box) and stored in the refrigerator (4 °C ± 1 °C) until further use. The refrigerated ears were removed from the refrigerator 2 h before application of the formulations, to enable temperature adjustment to room temperature. Group-D ears were frozen immediately (−20 °C ± 2 °C) after washing and drying. Approx. 12 h before use, the ears were removed from the freezer and placed in the refrigerator (4 °C ± 1 °C) for defrosting. Afterward, the same procedure was followed as for the fresh ears.

Before the application of the formulations, ears were fixed to polystyrene plates that were covered with aluminum foil. Intact skin areas (2 × 2 cm) without visible scratches and wounds were selected, marked and the hair within these areas was cut short (1-3 mm). On each test day, six ears were prepared this way. Ears 1–3 were used for the testing of the curcumin nanocrystals (Cur-NS and Cur-Gel), and the DiI-NLC and ears 4–6 were used for the testing of the smartFilms, Cur-NLC and CF-Gel (Table 3). The use of two independent ear sets was unavoidable due to space limitations, i.e., it was not possible to apply all six formulations on one ear. The setup enabled the testing of each formulation in triplicate. In addition, on each ear, the TEWL, skin hydration and skin friction were determined shortly before application of the formulations. The antioxidant capacity (AOC) of the porcine skin was determined for ears 4–6, respectively (Table 3).

The application of the formulations was performed according to previously established application protocols (Table 3). To minimize variations in application pressure and massage frequencies, which might cause changes in the penetration of the active compounds, the application of each formulation was performed with a saturated, gloved finger and by one individual only throughout the whole study. The formulations were allowed to penetrate the skin for 6 h in an oven with a temperature set to 32 °C ± 0.2 °C. After incubation, non-penetrated formulations were carefully removed from the surface of the skin with a soft tissue, and punch biopsies (Ø 15 mm) were taken from each test area. The skin biopsies were immediately embedded (Tissue-Tek^®^ O.C.T.™, Sakura Finetek Europe B.V., Alphen aan den Rijn, The Netherlands), frozen (−80 °C) and stored at −20 °C until further use.

#### 2.2.3. Determination of Penetration Efficacy

The dermal penetration efficacy was assessed from the punch biopsies according to a previously established protocol and by using a combination of epifluorescence microscopy with subsequent digital image analysis (EM-DIA) [44].

##### Epifluorescence Microscopy

Inverted epifluorescence microscopy was used to visualize and determine the penetration efficacy of curcumin and the fluorescent dyes from the different formulations. For this, 20 μm-thick vertical skin sections were prepared from the punch biopsies with a cryomicrotome (Frigocut 2700, Reichert-Junk, Nußloch, Germany) and subjected to inverted epifluorescence microscopy (Olympus CKX53 equipped with an Olympus DP22 color camera, Olympus Deutschland GmbH, Hamburg, Germany). The intensity of the fluorescence light source (130 W U-HGLGPS illumination system, Olympus Deutschland GmbH, Hamburg, Germany) and the exposure time were kept constant for each formulation to ensure comparability of the data. The fluorescence filter used was the DAPI HC filter block system with adequate excitation and emission filters for each respective formulation (Table 4). For statistical reasons, from each skin biopsy, at least 12 skin cuts were obtained and from each skin cut, at least three images were taken at 200× magnification. Hence, from each skin biopsy, at least 36 images were obtained. As each formulation was tested on three independent ears, meaning a total of at least 108 images was obtained for each formulation tested. The procedure was also performed for the untreated skin areas.

##### Digital Image Analysis

**Figure 2:**
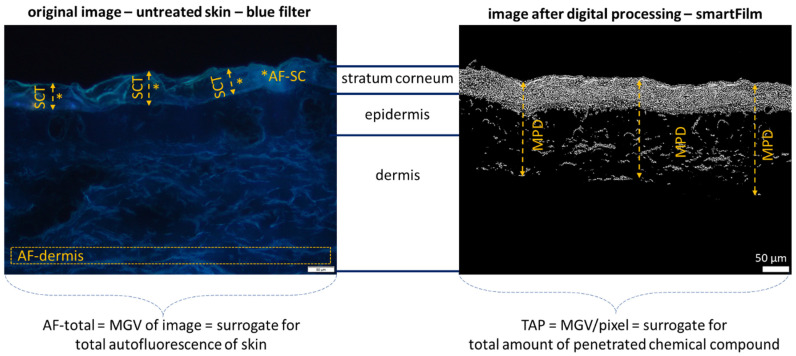
Microscopic images (magnification 200-fold) of untreated skin (**left**, unprocessed) and skin treated with curcumin-loaded smartFilms (**right**, after digital processing), visualizing the values obtained from digital image analysis. SCT = stratum corneum thickness, AF-SC = autofluorescence stratum corneum, AF-dermis = autofluorescence of the dermis, MPD = penetration depth of the chemical compound. In addition, the MGV of the whole images were measured. The MGV of the images of the untreated skin represents the autofluorescence of the whole skin (AF-total), and the MGV from the digitally processed images surrogates the total amount of penetrated chemical compound (TAP). SCT, AF-SC, AF-dermis and AF-total were assessed from the original images of untreated samples. TAP and MPD were obtained from digitally processed images treated with the respective formulations.

Digital image analysis (DIA) was performed with ImageJ software [44,45,46,47]. The epifluorescence images with 200× magnification were used to determine the penetration parameters, e.g., mean penetration depth (MPD) and the total amount penetrated (TAP, assessed as mean grey value/pixel). In addition, images were used to determine various skin parameters, i.e., thickness of the stratum corneum (SCT), the autofluorescence of the whole skin cut (AF-total), the AF of the dermis (AF-dermis) and the AF of the SC (AF-SC) (Figure 2—left).

The skin parameters (AF-total, AF-dermis, AF-SC, SCT) were assessed from the original images of the untreated skin areas. The SCT was determined with the scale bar of the ImageJ software after the scale was set to 2.84 pixel/µm. The AF-total is the mean grey value (MGV) of the image and the MGV is the average grey value (scale 0–255) within the image or image section, representing the sum of the grey values of all the pixels divided by the number of pixels [48]. High MGV, therefore, correspond to high brightness, and low values correspond to low brightness. Consequently, low MGV represent a low AF of the skin. To distinguish the AF of the dermis and the SC, the MGV of the SC was determined from the lines used for SCT measurements (Figure 2—left), and the AF of the dermis was determined from image sections of constant position and size from the lower part of the images (Figure 2—left, Supplementary Material Section S1).

The penetration efficacy of the different compounds from the different formulations was assessed as MPD and TAP, which were determined from images that were first subjected to an auto-threshold algorithm to minimize the AF of the skin from the image (Figure 2—right, cf. Supplementary Material Section S2 for a detailed description of threshold-algorithms). After elimination of the skin AF, the remaining bright pixels within the images corresponded to the total amount of penetrated compound into the skin [44]. Therefore, the MGV of the image after automated thresholding was determined and was used as a surrogate for the total amount of penetrated compound (TAP); the penetration depth was also determined by measuring the penetration depth in each autofluorescence corrected image (Figure 2—right).

#### 2.2.4. Determination of Biophysical Skin Properties

The biophysical skin parameters TEWL, skin hydration, skin friction and skin stiffness were assessed with the Multi Probe Adapter MPA 10 device (Courage & Khazaka electronic GmbH, Cologne, Germany), to which the required skin probes Tewameter^®^ TM 300, Corneometer^®^ CM 825, Frictiometer FR 700 and Indentometer IDM 800 (all from Courage & Khazaka Electronic GmbH, Cologne, Germany) were connected. Measurements were performed at least in triplicate, at room temperature (22 °C ± 1 °C) and in a room with a relative humidity of 40–63%.

The AOC of the skin was determined from the SC that was removed from the skin via the classical tape-stripping procedure [49,50]. SC was collected with 30 tapes (Tesafilm^®^ crystal clear, tesa SE, Beiersdorf AG, Norderstedt, Germany). Subsequently, the SC stuck to the tapes was extracted in 5 mL 70% ethanol under shaking at 160 rpm for 1 h (Edmund Bühler Swip KS-10, Edmund Bühler GmbH, Bodelshausen, Germany). From the SC extracts obtained, the AOC was determined using an oxygen radical absorbance capacity (ORAC) assay [51,52]. For this, 20 µL of SC extract was added to the cavity of a black 96-well plate, along with 150 µL of 1 µM fluorescein (Alfa Aesar, ThermoFisher GmbH, Kandel, Germany) in phosphate-buffered saline (PBS, pH 7.4). The plate was incubated at 37 °C for 15 min, and then 90 µL of AAPH (125 mM, 2,20-azobis(2-methylpropionamidine) dihydrochloride, Acros Organics, Geel, Belgium) in phosphate-buffered saline (PBS, pH 7.4) was added rapidly to each well. The fluorescence intensity of the sample was then determined every minute for 100 min (FluoStar^®^ Optima, BMG Labtech, Offenburg, Germany, excitation wavelength: 485 nm, emission wavelength: 520 nm). Trolox (6-hydroxy-2,5,7,8-tetramethylchroman-2-carboxylic acid, Santa Cruz Biotechnology Inc., Dallas, TX, USA) served as the external standard in concentrations from 22.2 to 100 µM in PBS (pH 7.4). The AOC was calculated in regard to the calibration curve of Trolox concentrations versus the net area under the curve of the fluorescence decay curves. The results were expressed as µM Trolox equivalent (µM TE).

#### 2.2.5. Statistical Analysis

Statistical analysis was performed with JASP software version 0.13.1(University of Amsterdam, Amsterdam, The Netherlands) [53]. Shapiro–Wilk tests were performed to check for normal distribution of the data, Levene tests were performed to check for variance homogeneity and outliers were identified by the interquartile rule [54]. Parametric data were subjected to one-way analysis of variance with Welch adoption in case of variance inhomogeneity. Non-parametric data were subjected to Kruskal–Wallis H tests. Tukey, Games–Howell or Dunn’s post-hoc tests were performed to determine significant differences between the mean values. Bonferroni–Holm adjustment was done to account for alpha error accumulation [55]. Descriptive graphs represent the mean values ± SD obtained from the skin biopsies. Statistical relationships between the different parameters were investigated by determination of the respective correlation coefficients (Pearson’s correlation coefficient r for normally distributed data, Spearman’s rank correlation coefficient ρ for non-parametric data). Probability values (*p*-values) < 0.05 were considered as statistically significant.

## 3. Results

### 3.1. Influence of Post-Slaughter Pig Ear Age on Dermal Penetration Efficacy of Chemical Compounds

**Figure 3:**
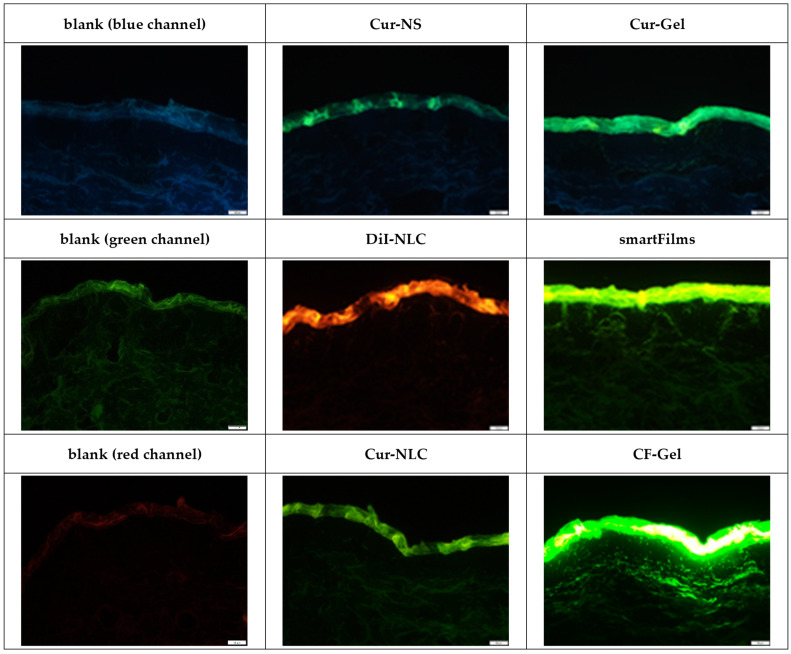
Microscopic images of skin sections treated with different formulations (cf. Table 2) obtained by inverted fluorescence microscopy (magnification 200-fold, scale bar = 50 µm).

The observation of the original images obtained from epifluorescence microscopy revealed—as expected—pronounced differences in the penetration efficacy (MPD, TAP) between the different formulations (Figure 3). A very pronounced transdermal penetration was found for CF from the collagen gel (CF-gel), and also, the smartFilms enabled a very pronounced transdermal penetration of curcumin. A less pronounced penetration was found for curcumin from the NLC (Cur-NLC) and from the nanocrystals (Cur-NC and Cur-Gel). DiI was found to penetrate efficiently into the stratum corneum from the NLC, and transdermal penetration was observed randomly in some of the biopsies.

**Figure 4:**
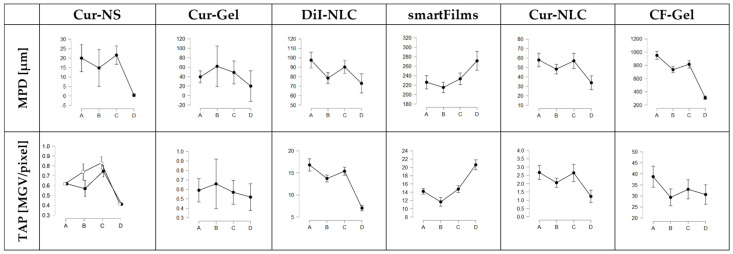
Influence of porcine ear age on penetration efficacy for the six different formulations tested (cf. Table 2). (A) = fresh ears, (B) = ears stored for 24–36 h, (C) = ears stored for 48–60 h, (D) = frozen and on-demand defrosted ears. Black dots represent data with outliers included. White dots represent means after elimination of outliers. Lines between the different post-slaughter ages represent the changes in penetration efficacy based on the storage time of the ears.

Despite the differences in penetration efficacy between the different formulations, the penetration efficacy was also affected by the post-slaughter age of the pig ears (Figure 4). The data obtained possessed a broad biological variety (cf. Supplementary Material Section S3), but the averaged results show a clear trend (Figure 4). The trend was not linear, because the use of B-ears (ears stored for 24 h before use) resulted in a less efficient penetration than the use of the A-ears (fresh ears) or C-ears (ears stored for 48 h before use). The trend was observed for both penetration parameters (MPD and TAP) for the Dil-NLC, smartFilms and Cur-NLC. For Cur-NS, the trend was observed for the MPD and also became visible for the TAP of the Cur-NS after the outliers were excluded (Figure 4—A ears; (lower)—black vs. white dotted lines). The Cur-Gel revealed a higher penetration for curcumin when B-ears were used (Figure 4—B ears). However, the higher penetration of the Cur-Gel when B-ears were used was caused by only two ears that led to extremely high penetration values, even though they were not identified as outliners. After exclusion of these two ears from further analysis, the Cur-Gel data also followed this trend (cf. Supplementary Material Section S4). The differences in penetration efficacy between the differently aged ears were significant for all formulations, and post-hoc tests showed that, in most cases, differences were only significant for two ear types. Hence, significant differences were either found between A and B ears, between A and C ears or between B and C ears. Frozen and defrosted ears (D ears) resulted in a significantly less efficient penetration when compared to fresh ears (Figure 4). Here, one exception was also observed, because the penetration efficacy of curcumin from smartFilms was increased when frozen and defrosted ears were used (Figure 4—D ears).

The opposite penetration of curcumin from smartFilms on the frozen and defrosted ears might indicate a different penetration mechanism of curcumin from the smartFilms. Indeed, this is reasonable, because the smartFilms were the only formulation that was applied as a patch, whereas all other formulations were applied as liquid or semisolid formulation.

The all-over observed “up and down” effect in penetration efficacy that occurred with increasing post-slaughter age of the ears was not conclusive, but the significant differences that were observed indicated that—in addition to post-slaughter age—other parameters also seem to contribute to the penetration efficacy. Possible parameters that affect the penetration efficacy of chemical compounds are the skin properties, e.g., skin hydration and/or TEWL. These parameters are considered sensitive markers for the integrity of the skin barrier and thus were also assessed in this study.

### 3.2. Influence of Post-Slaughter Pig Ear Age on Skin Properties

**Figure 5:**
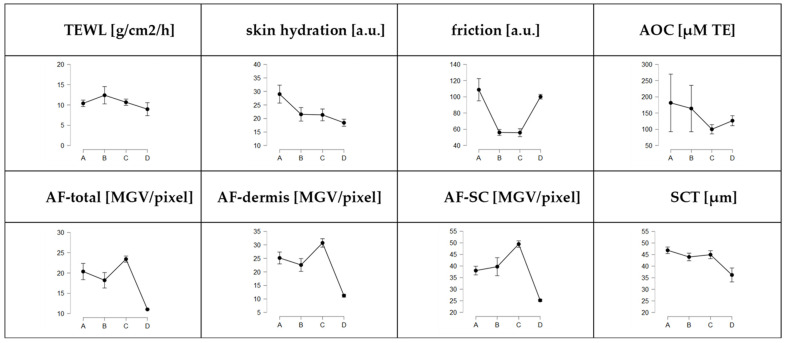
Influence of storage time (age) on skin parameters. A = fresh ears, B = ears stored for 24–36 h, C = ears stored for 48–60 h, D = frozen and on-demand defrosted ears.

**Figure 6:**
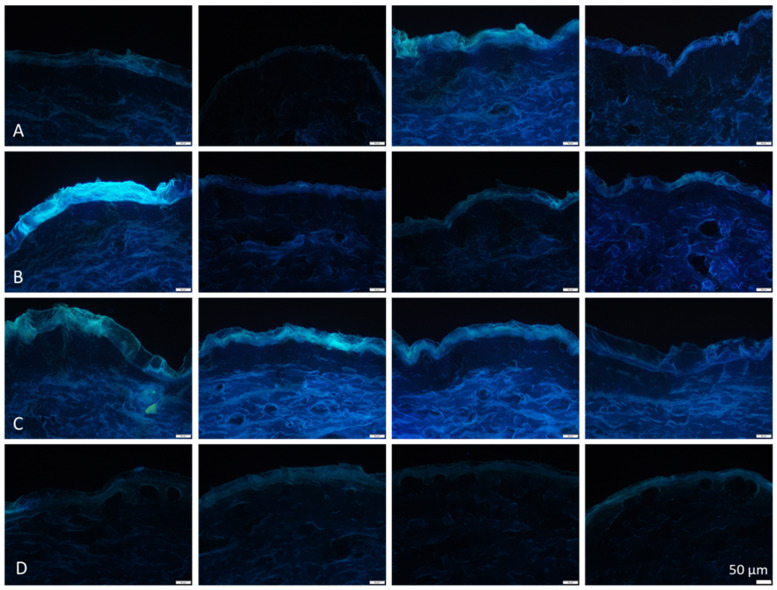
Selected microscopic images from differently aged blank skin biopsies (non-treated skin sections) obtained by inverted fluorescence microscopy (magnification 200-fold, scale bar = 50 µm). (**A**) = fresh ears, (**B**) = ears stored for 24–36 h, (**C**) = ears stored for 48–60 h, (**D**) = frozen and on-demand defrosted ears. Four images were selected for each post-slaughter age to visualize the pronounced variations in skin autofluorescence between the ears.

The post-slaughter age of the pig ears also affected the skin properties of the ears, indicating the ears undergo post-mortem changes (Figure 5). In addition, other skin parameters, i.e., SCT, AF of the skin, AOC and skin friction, were assessed. These values were also found to change with increasing post-slaughter age of the ears (Figure 5 and Figure 6).

The observation of the microscopic images showed pronounced variations in skin autofluorescence between the ears (Figure 6). In particular, the fresh ears (A-ears) possessed pronounced differences in the AF. Interestingly, despite biological variations, data from digital image analysis demonstrated a similar “up and down” trend for the skin parameters (Figure 5) as that already seen for the penetration data (cf. Section 3.1). Hence, the AF of the skin and the SCT were lower for the B-ears than for the A- and C-ears, respectively. In contrast, the TEWL was higher for the B-ears and lower for the A- and C-ears. In the case of frozen and on-demand defrosted ears, skin hydration, friction and AOC were found to decrease with increasing storage time of the ears, and freezing resulted in lower AOC values than fresh ears but was not as low as that observed for the C-ears (Figure 5). The other skin parameters, i.e., AF, SCT, TEWL and hydration, were significantly decreased when compared to the fresh ears and skin friction was significantly increased (Figure 5).

The trends seen for the different skin properties from the averaged results of the fresh ears were not seen for all ears, and for some ears, opposite effects were even observed. For example, instead of a decrease in skin parameter value, an increase in the skin parameter after one day of storage was seen for some ears. However, in these cases, a decrease in skin parameter values was seen the next day. Hence, the “up and down” effects seemed to occur in all ears, but the time points at which the fluctuation occurred seemed to be different. Due to the sometimes-opposed effects, the “up and down” trend observed was not significant for the whole set of data, but differences between the different days of storage became significant when the data for each trial (cf. Table 2) were compared to each other individually. Due to the significance of the data for both the skin parameters and the penetration data, the observed “up and down fluctuations” were considered as real effects. The assumption that the fluctuations in skin properties and penetration efficacy are real raised the question of whether there are known post-mortem phenomena that might cause the observed effects.

Indeed, literature screenings revealed that a well-described effect in the meat industry that causes fluctuations in meat properties is post-mortem contraction, also known as post-mortem rigidity or rigor mortis. This occurs in pigs within 4–6 h after slaughtering [56]. Rigor mortis occurs because, after slaughter, blood circulation in the pigs stops, and hence, oxygen is no longer provided. This causes the aerobic metabolism to change into anaerobic metabolism. In addition, calcium enters the cytosol after death due to the deterioration of the sarcoplasmic reticulum. The calcium activates the formation of actin-myosin cross-bridging and triggers the contraction of the entire muscle fiber. The relaxation of the muscle requires adenosine triphosphate (ATP), which can be provided via anaerobic glycolysis after slaughter. For this, glycogen is converted to lactic acid, which causes a decrease in pH. When the pig’s glycogen is depleted, the ATP concentration decreases. If ATP falls below a critical level, it is unable to break the actin-myosin bridges, and consequently, the carcass enters rigor mortis [57,58]. The actin-myosin cross-bridging results in a shortening of the muscle. This reduces the intercellular space in the tissue, and with this, the water holding capacity. This means that the onset of rigor mortis causes the tissue to expulse water, which results in a moist surface and tissue with a lower water content [58,59]. The decrease in pH—due to the accumulation of lactic acid—further reduces the water holding capacity because it shifts the pH of the tissue more toward the isoelectric point (IP) of the proteins. This results in a decreased polarity (charge) of the proteins and causes a lower binding capacity of the polar water molecules [60,61]. Hence, carcasses in rigor mortis contain less water. Rigor mortis in pigs lasts for 24–48 h and is then resolved due to the lysis of proteins. The muscles become soft and tender, which results in an increase in the water-holding capacity [60,61].

The onset and duration of rigor mortis after slaughter can be very variable. Hence, some pigs develop rigor mortis more quickly and more intensely than others. The reasons are manyfold and include the race, physical state and age of the pig. The type of food, the stress before slaughter and also the storage temperature of the carcasses have a tremendous influence on the onset, intensity and duration of rigor mortis. Pigs that were stressed shortly before slaughter release greater amounts of glycogen, which can then be quickly broken down to lactic acid. The fast decline in ATP and the fast reduction in pH cause a faster onset of the rigor mortis and a fast and pronounced expulsion of the water from the muscle (meat of such quality is termed “pale, soft and exudative (PSE) meat”). In contrast, if the pigs were extremely exhausted before the slaughter, e.g., due to long transportation, the glycogen level was low. The reason for this is that the released glycogen was converted to lactic acid when the pigs were still alive. Due to the still-existing blood circulation at that time, the lactic acid was not accumulated in the muscle fibers but instead transported and metabolized in the liver. Meat of this kind is, therefore, low in ATP, has no glycogen as a reservoir and will produce less lactic acid post-mortem than normal meat. Meat of such kind is known as “dark, firm and dry (DFD) meat” and is characterized by a high pH and very high water-binding capacity [57,59].

The data obtained in this study, therefore, suggest that the “up and down” effect in skin properties and penetration efficacy is caused by rigor mortis of the porcine ears. This means that pig ears undergo post-mortem contraction. The described decrease in water-binding capacity during rigor mortis and its increase after rigor mortis are indicated by an increase in TEWL and a decrease in SC-thickness for the B-ears, followed by an increase in SC-thickness and a decrease in TEWL for the C-ears (Figure 5). Various studies substantiate this assumption. For example, changes in skin hydration are also described to affect the AF of the skin, i.e., a lower skin hydration results in a decreased AF of the skin [62,63]. Hence, the observed decrease in AF can be explained by a decrease in hydration due to rigor mortis. In addition, it can be explained by a decrease in NAD(P)H, which occurs due to necrosis of the tissue [64,65]. The water content of the skin is known to have an extreme influence on the penetration efficacy of chemical compounds. Therefore, in light of rigor mortis, the observed decrease in penetration for the ears that were stored for 24 h before use (B-ears) becomes highly reasonable. The lower water content in the skin reduces the intercellular space in the skin tissue, and in addition, the expulsed water can be considered to create a push effect toward the outside of the skin, thus further reducing the flux of chemical compounds into the skin.

Longer storage of meat causes rigor mortis to be released. This is caused by enzymes (proteases) that cause the cleavage of skin proteins. Proteolysis mainly affects the collagen structure and thus causes softening of the tissue and a pronounced release of amino acids. The main amino acid that is released at this stage in type 1 collagen is tyrosine, which possesses strong autofluorescence [66]. Consequently, and as seen in the present study, an increase in free tyrosine will cause an increase in AF. As the effect occurs in the viable dermis, the effect is mainly seen by the increase in the AF of the dermis (Figure 6). The release of free amino acids also causes an increase in pH. This increases the net charge of the tissue, and therefore, leads to an increase of the water-holding capacity [67,68]. The increase in skin AF and hydration was also seen in the present study and can explain the increased penetration efficacy for the C-ears.

In fact, data obtained in this study fully support the theory that pig ears undergo rigor mortis during storage. These changes in properties that occur due to rigor mortis affect the skin properties of the porcine ears and affect the penetration efficacy of chemical compounds when tested on ears with different post-slaughter age. It must be emphasized that rigor mortis occurs at very different and non-predictable time points—mainly depending on the pre-history of the pig before slaughter.

### 3.3. Evaluation of Rigor Mortis Theory

Rigor mortis in carcasses and its influence on meat properties is well-described in the literature but no data are available on the occurrence of rigor mortis in porcine ears. Therefore, to prove the rigor mortis theory in pig ears, skin parameters, i.e., TEWL, skin hydration, skin firmness, pH, thickness and weight, were assessed at different time points after the collection of the ears. As rigor mortis is known to occur in pigs at about 4–6 h after slaughter and to last for 24–48 h, parameters were assessed not only shortly after the collection of the ears but also at 12 h, 24 h, 36 h, 48 h and 60 h after collection of the ears. In addition to the previously assessed skin data (cf. Section 3.2), to enable the detection of increasing skin stiffness due to the onset of rigor mortis, the skin firmness was determined with an intendometer [69].

**Figure 7:**
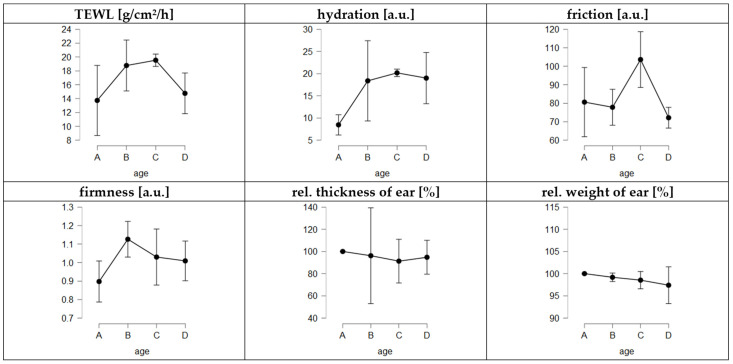
Influence of storage time of ears on skin parameters. A = fresh ears (4–6 h after slaughter), B = ears 10–12 h after slaughter, C = ears 24–36 h after slaughter, D = ears 48–60 h after slaughter.

The results obtained show a higher skin firmness for the ears after 12 h, which decreased during storage (Figure 7). Thus, we provide the first evidence that rigor mortis in porcine ears occurs during the first hours after slaughtering. The increase in skin firmness was associated with an increase in skin hydration and TEWL and with a decrease in skin friction, ear thickness and weight (Figure 7). The data thus show that the onset of rigor mortis is associated with an expulsion of water from the ear. The elevated TEWL is an indication of accelerated water evaporation from the ear, which results in a higher water content on the skin. This can be seen by greater skin hydration and reduced skin friction, i.e., the water on the skin causes it to be less sticky than dry skin. The reduced water content also causes a reduction in the weight and thickness of the ear.

Rigor mortis started to be released after 24 h (decrease in skin firmness), but the water retention seemed to remain low for a longer period. Between 24–36 h after slaughter, the TEWL remained high and the skin thickness and weight were further reduced, which indicates water loss and drying of the skin. The skin friction was very high after 24–36 h of storage, indicating that the surface of the skin was now very dry, thus causing a highly sticky skin surface. After further storage, the TEWL and skin hydration decreased. The skin friction decreased, and the thickness of the ears increased. Hence, proteolysis and the release of rigor seem to come into effect after about 48–60 h of storage (Figure 7). Based on the data, we conclude that pig ears undergo post-mortem changes that are comparable to the effects well-described for carcasses.

This phenomenon now explains the large variations in the penetration results from this study (cf. Figure 4). The onset and intensity of rigor mortis depend on the energy level of the pig before slaughter (cf. Section 3.2), and the pig ears used in this study can be expected to have been derived from pigs with different stress levels. Pigs that were stressed shortly before slaughter were more likely to provide ears with PSE-like properties, and pigs that were exhausted for a longer period before slaughter were more likely to provide ears with DFD-like properties.

Despite some very recent results that already provide evidence that pre-slaughter stress, i.e., due to an elevated temperature in summer, can affect the properties (antioxidative capacity) of ex vivo porcine ears [70], no literature is available on the influence of pre-slaughter stress on the properties of porcine ears. However, based on literature from food-processing technology, one can expect that the onset of rigor in PSE-like ears will be faster than in normal ears and less intense in DFD-like ears. Consequently, PSE-like ears should possess lower pH values and higher skin hydration directly after collection from the slaughterhouse and—due to the fast and more intense onset of rigor—this should lead to a less efficient penetration of chemical compounds when compared to normal ears. DFD-like ears are expected to possess a high water-holding capacity. Thus, DFD-like ears are expected to yield extremely high penetration values. The effect is expected to occur on fresh ears (A-ears) and is probably more pronounced when ears that are stored for 24–36 h (B-ears—ears that are typically in rigor mortis) are used for penetration studies. In this type of ear, the difference in penetration efficacy can be expected to be most pronounced because normal ears are in rigor mortis and lead to low penetration of active compounds, whereas DFD-like ears are fully hydrated, thus allowing for good penetration of active compounds into the skin.

**Figure 8:**
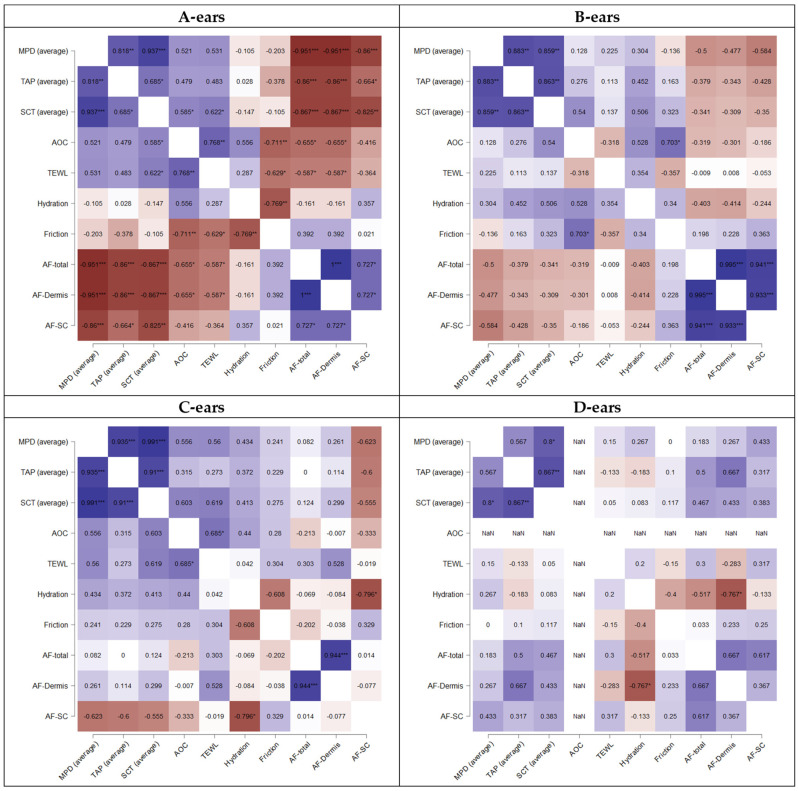
Correlation of penetration values and skin parameters (Spearman’s rho). Explanations cf. text. * *p* < 0.1, ** *p* < 0.01, *** *p* < 0.001.

Consequently, it was expected that pH and skin hydration, when assessed from fresh ears, might be sensitive markers to identify PSE and DFD-like ears. The pH values were not assessed in this study, but skin hydration, skin friction and TEWL were analyzed. These data were, therefore, used to investigate whether the penetration efficacy (MPD, TAP) and skin properties (TEWL, skin hydration and friction) correlated with each other. However, no statistical relationship between skin properties and penetration data was observed (Figure 8).

The outcome was not expected, but is indeed reasonable, because high skin hydration and/or high TEWL values are typically associated with an improved dermal penetration efficacy. In contrast—as seen in this study—rigor mortis results in similar skin properties (high TEWL, high skin hydration) but leads to an opposite penetration efficacy. Hence, ears with high TEWL and/or high skin hydration values can represent both, i.e., ears with either good or poor penetration. Based on the data, it must, therefore, be concluded that the measurement of TEWL and skin hydration alone is not sufficient to determine the condition of pig ears efficiently. However, to date, especially these parameters are often used to identify suitable skin areas on the ears that are intended for use in ex vivo penetration studies [71,72,73,74,75,76,77,78]. Hence, in the future, other and/or additional parameters need to be assessed to securely distinguish between ears with good or poor penetration efficacy and to allow for a more meaningful selection of suitable, intact skin areas.

One suggested additional parameter is the assessment of the pH of fresh ears. Low pH values would identify PSE-like ears, and high pH values—being associated with low skin hydration—would identify DFD-like ears. Despite the pH, the data of this study also suggest that the AF of the skin might be a sensitive marker to discriminate normal ears from ears that lead to poor or too-high penetrations of chemical compounds. The assumption is based on the fact that the AF of the skin was negatively correlated with the penetration efficacy (Figure 8A). Hence, ears with high AF were associated with a low penetration efficacy (MPD, TAP) and vice versa. The correlation was significant for fresh ears (A-ears) and declined with the increasing post-slaughter age of the ears (Figure 8). The observed effects can be explained by the different parameters that affect the AF of the skin and by the different post-mortem stages that occur after slaughter.

The AF of the skin is known to be influenced by various parameters [64]. One parameter—especially when observed in the blue channel—is the amount of NAD(P)H, where high levels of NAD(P)H are associated with a high AF [64,65]. Another parameter is the skin hydration, and also here, high hydration of the SC is associated with a high AF-SC [63]. Typically, the NAD(P)H depletes when tissue or cells undergo metabolic death, which results in a constant decrease in AF. However, recent studies also observed an increase in autofluorescence on organ death that occurred before the expected decrease in AF took place [79]. The effect, sometimes referred to as the AF wave [79], was also observed in excised human skin [65] and was recently linked to the onset of rigor mortis in c. elegans [80]. In the present study, the onset of rigor was shown to cause an increase in skin hydration and TEWL, which can also be expected to cause an increase in AF. Hence, a high AF of fresh ears can be considered to pinpoint ears with early onset of rigor mortis and less penetration efficacy (PSE-like ears). DFD-like ears can be expected to contain less NAD(P)H, and despite a high water-holding capacity within the tissue, a dry SC. This would lead to a low AF, and hence, ears with very low AF can be expected to possess DFD-like properties with a corresponding high penetration efficacy for chemical compounds. Therefore, the observed negative and significant correlation between AF and the penetration efficacy for fresh ears (A-ears) is reasonable and suggests that the AF is a meaningful skin parameter for the selection of suitable and non-suitable fresh pig ears for ex vivo penetration studies.

Once the ears are in rigor, the water content declines and the NAD(P)H in the necrotic tissue declines. This explains the observed decrease in AF, and also, the loss in the correlation between AF and penetration efficacy found for fresh ears (Figure 8B). The reason is that all ears in rigor possess a poor penetration, which is now associated with low AF, and not to a high AF, as seen for the fresh ears. The release of rigor leads to a release of free amino acids and causes changes in the pH. The water content is increased, and the AF is increased due to the increased water content. In addition, proteolysis of the collagen results in the release of tyrosine, which also results in an increase in AF (mainly in the dermis). This explains the higher AF found for the C-ears. In post-rigor, a high AF is, therefore, associated with a good penetration efficacy. The expected trend is indeed visible in the heatmaps of the correlated data. However, it is not yet significant (Figure 8C). Frozen and defrosted ears possess a low AF (Figure 5 and Figure 6). This is caused by low levels of NAD(P)H that depleted during storage in the freezer and by the absence of post-rigor changes, i.e., the proteolysis of the connecting tissue. In addition, the SC of the defrosted ears is dehydrated (thinner SCT, reduced TEWL, low skin hydration, high friction—cf. Figure 5), thus further contributing to a low AF of the frozen and defrosted ears. Defrosted ears with greater hydration can be expected to possess a higher AF and can also be considered to allow for a better penetration of chemical compounds. Therefore, the trend between AF and penetration is the opposite of that of fresh ears, i.e., ears with high AF are associated with a higher penetration efficacy (Figure 8D).

### 3.4. Identification of Most Suitable Post-Slaughter Age for Porcine Ears

Based on the good fit between the data obtained in the present study and the theoretical facts that are known about post-mortem changes from other disciplines, it was interesting to see if data from this study would help to identify ears with exceptional penetration properties. For example, DFD-like ears can be expected to lead to the most effective penetration because rigor mortis is weaker than for normal ears. Hence, ears will not undergo dehydration during rigor that might be indicated by a low skin hydration. These ears will also lack the rigor-associated AF wave. Hence, such ears should possess a much lower AF than other ears with similar post-slaughter ages.

In this study, there were two ears that resulted in significantly better penetration results than all other ears. These ears were, for example, responsible for the higher detected penetration of curcumin from the Cur-Gel for the B-ears, whereas exclusion of these data resulted in a trend seen for all other formulations (less penetration from B-ears, cf. Section 3.1). Indeed, these two ears possessed the lowest AF from all ears (MGV/pixel < 15) and also belonged to the group of ears with the lowest skin hydration (corneometer values < 20), thus providing the first evidence that the AF can be used as an additional parameter for the selection of suitable porcine ears for ex vivo penetration experiments. Interestingly, the TEWL values of these ears were unremarkable and were in the range between 10 and 10.5 g/h/cm^2^. The data, therefore, also substantiate the finding that TEWL measurements alone are not sufficient to identify ears with “non-normal” skin penetration properties.

**Figure 9:**
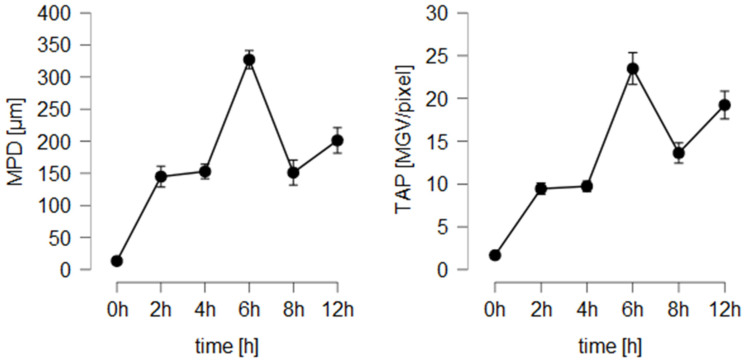
Influence of penetration time on penetration efficacy of curcumin from smartFilms.

The fact that rigor mortis in pig ears affects the penetration efficacy was not yet considered in previous studies. Therefore, the new findings require a thoughtful re-evaluation of the use and possible applications of the ex vivo porcine ear model. So far, in the literature, ears that are freshly obtained from the slaughterhouse and directly used on the day of slaughter have been considered to be the most appropriate ears for dermatopharmacokinetic studies. However, the new data indicate that the properties of fresh ears—due to differences in rigor mortis onset—can possess a large biological diversity that can change quickly during the first hours after slaughter. As these post-mortem changes cause a reduction in penetration efficacy, it can be assumed that—due to the push effect of expulsed water from the ear due to the onset of the rigor—the penetration efficacy of a compound might decrease while running a longer-lasting penetration experiment. In this case, the penetration kinetics would change, thus leading to non-linear, distorted penetration profiles. The theory was tested by investigating the penetration efficacy of the smartFilms on freshly obtained porcine ears over a longer period (12 h) and by collecting skin biopsies at different time points (Figure 9).

The results confirm an increase in dermal penetration within the first 6 h, but a decreased penetration efficacy of curcumin after 8 h and 12 h. The data, therefore, demonstrate that the onset of rigor causes a reduction in the penetration efficacy, thus leading to non-linear penetration profiles. The use of fresh porcine ears is, therefore, critical for running dermatopharmacokinetic studies where the penetration efficacies of different time points are compared to each other. Hence, such studies probably require the use of internal standards, careful assessment of the skin parameters throughout the whole experiment and thoughtful interpretation of the data.

Aside from the large variations that occur in the fresh ears due to the large variations in the onset of rigor mortis, the use of ears on the same day of slaughter is not always convenient. The transportation, preparation and skin analysis of the ears are time-consuming, which means that the penetration experiments cannot start early in the morning. As a consequence, the risk of an onset of rigor mortis during the experiment increases and, in addition, long working days are needed to finish the experiments. The use of ears that were prepared for the penetration experiments in advance, i.e., one day before the penetration experiments start, allows to conduct the penetration experiments itself more effectively. Therefore, in daily practice, the use of post-slaughter stored ears is much more convenient than the use of fresh ears without post-slaughter storage. In this light, it was interesting to evaluate, if the use of post-slaughter aged ears is also feasible. 

**Figure 10:**
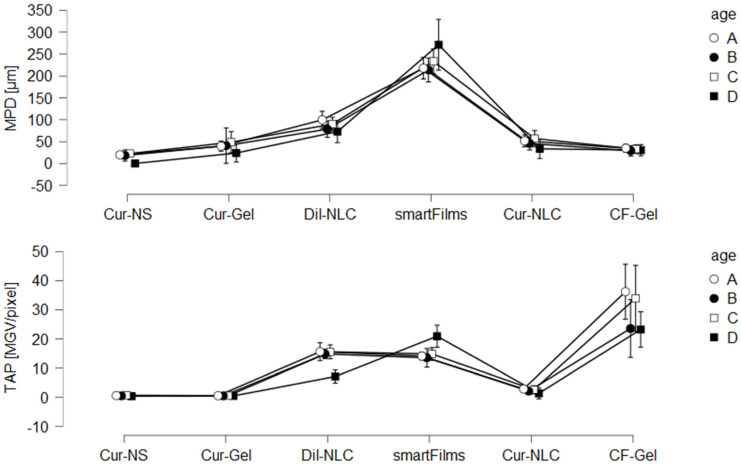
Comparison of influence of pig ear age (A = fresh ears, B = ears stored for 24 h before use, C = ears stored for 48 h before use, D = freshly frozen ears and on-demand thawing) on dermal penetration efficacy (MPD and TAP).

When comparing the absolute penetration values obtained from the different formulations and the differently aged ears, one can summarize that the ex vivo penetration model coupled with EM-DIA of the skin biopsies proved to be a sensitive tool for assessing the dermal penetration efficacy of fluorescent compounds. With this, it was possible to detect even small differences between the formulations and between ears with different post-slaughter ages. A rough estimation of the influence of post-slaughter age on the penetration efficacy of chemical compounds was carried out by setting the averaged penetration values of fresh ears (no post-slaughter storage) to 100% and by comparing the absolute values obtained from the other ears to these values. Data show that 24-h post-slaughter storage reduces the penetration efficacy by about 15%. Post-slaughter storage for 48 h revealed a slightly higher penetration than fresh ears (approx. +5%), and frozen and defrosted ears reduced the penetration efficacy by about 30%. Due to this, it is not possible to directly compare the absolute penetration values obtained from ears with different post-slaughter ages. However, a rough trend, i.e., a rough estimation between good and bad penetrating formulations, seems to be possible when comparing the results obtained from ears with different post-slaughter age. Hence, for the six different formulations tested, the age of the ears did not influence the ranking of the penetration efficacy between the different formulations. This means, independent of the post-slaughter age of the ear, a similar trend, i.e., a penetration efficacy of CF-Gel > smartFilms > DiI-NLC > Cur-NLC > Cur-Gel = Cur-NS, was observed (Figure 10).

Due to the similarity in the ranking of the different formulations, it is concluded that not only freshly obtained ears but also ears that are stored for 24 h or 48 h after slaughter can be used for ex vivo penetration studies. Moreover, frozen and on-demand defrosted ears were confirmed to be suitable for utilization in ex vivo penetration studies. All these ears led to similar penetration trends between the different formulations. Moreover, all types of ears showed a positive correlation between the SCT of the untreated skin and the penetration efficacy of the formulations (Figure 8). Hence, the influence of the SCT on the penetration efficacy can also be investigated in all differently aged ears. The use of frozen and on-demand defrosted ears is convenient because a slaughterhouse nearby is not required. Hence, fresh ears can be collected, frozen and stored until needed. This procedure is most convenient and thus might be suggested to be the most suitable option if the ex vivo model is used to compare the dermal penetration efficacy of chemical compounds from different formulations.

The lower penetration efficacy from frozen and defrosted ears is due to the dehydrated stratum corneum (cf. Figure 5). With this, the stratum corneum is tighter, which results in a less efficient penetration for chemical compounds. Albeit, the data obtained from the smartFilms suggest that the penetration-reducing effect might be circumvented if the SC can be rehydrated. In this case, the dermal penetration is expected to improve and become even more effective when compared to non-frozen ears because freezing destroys the structure of the viable dermis, thus allowing for an increased flux of the chemical compounds into the skin. The effect is, therefore, expected to be most pronounced for formulations with pronounced transdermal penetration.

The theory is already supported by the penetration data obtained from the smartFilms. The smartFilms were applied in a wet state and were covered with a patch that prevented drying out of the smartFilms. Of course, it is highly likely that the patch also caused an effective rehydration of the SC, which then caused the observed increase in the penetration efficacy of curcumin from the smartFilms on frozen and defrosted ears (Figure 4). The data, therefore, suggest that the dehydration of the frozen and defrosted ears is reversible. Hence, it can be assumed that rehydration of the SC before penetration experiments can increase the dermal penetration. Further research and the development of a protocol for optimal rehydration of the SC before penetration testing are now needed to further improve and validate this freeze-thaw ex vivo penetration model.

### 3.5. Comparison of Penetration Efficacy of Curcumin from Different Vehicles

The study was conducted with four different formulation strategies and thus it was also possible to compare the efficacy of the different formulations strategies to transport the poorly soluble compound curcumin into and/or through the skin. Despite the different formulation principles (aqueous nanosuspension, nanosuspension in gel with humectant, NLC and smartFilms, cf. Table 1), each formulation contained different amounts of curcumin. Due to this, also different amounts of curcumin were applied on the skin (Table 3 and Table 5). 

The determined TAPs for Cur-NS and Cur-Gel were identical but the MPD was more than doubled for the Cur-Gel (Table 5). With this, the here observed increase in penetration efficacy for the Cur-Gel supports previous findings, were the influence of the vehicle on the dermal penetration efficacy of curcumin from a nanosuspension was investigated.

In this study, it was shown that xanthan-gum in the Cur-Gel acts as moisturizer, which prevents a drying out of the formulation during incubation. This causes the SC to swell, thus leading to an increase in the dermal penetration efficacy [40]. The curcumin applied on the skin with the smartFilms was only 13.7% of that applied with the Cur-NS but resulted in a 26-fold increase in penetration efficacy (TAP, Table 5). This improved penetration of curcumin from smartFilms when compared to curcumin bulk- and nanosuspensions was also shown recently [42]. The data of the present study could, therefore, fully confirm these results and substantiate that dermal penetration of curcumin can be enhanced when curcumin is loaded in a cellulose fiber matrix in amorphous state. A comparison of the dermal penetration efficacy of curcumin between a curcumin nanosuspension and an NLC formulation is not yet available in the literature. However, such a comparison is of high interest because both formulation principles are well-recognized to enable an improved dermal penetration of poorly soluble compounds [81,82,83,84,85,86,87]. The NLC formulation used in this study contained very low amounts of curcumin (250-fold lower when compared to the NS) but the total amount penetrated was about four-fold higher (TAP, Table 5). In fact, smartFilms—and especially the NLC formulation—were found to be superior formulation strategies for efficient passive dermal and transdermal penetration of curcumin.

## 4. Conclusions

The ex vivo porcine ear model coupled with epifluorescence microscopy and subsequent digital image analysis was confirmed to be a sensitive and suitable tool for assessing the dermal penetration efficacy of fluorescent compounds. Most important, the study revealed that porcine ears undergo post-mortem changes. These changes can be linked to rigor mortis and all other well-described phenomena that occur with carcasses after slaughter. Rigor mortis affects the skin properties of the ears and the penetration efficacy of chemical compounds. This needs to be considered when assessing the dermal penetration efficacy with the ex vivo porcine ear model, especially when comparing data from different ear studies. During rigor mortis the penetration efficacy is reduced, and it increases once the rigor is released. Therefore, data obtained from ears with different states in rigor cannot be compared to each other directly.

Despite the differences in the absolute penetration values, the post-slaughter ages of ears did not affect the ranking of the penetration efficacy from the different formulations. Therefore, our data suggest that ex vivo penetration testing can be done either with fresh ears, with ears post-mortem stored for up to 48 h or with frozen and on-demand defrosted ears. Each type of ear has advantages and disadvantages, and the differences within these ears might even provide the possibility to develop sensitive ex vivo models for different skin conditions and/or diseases in the future.

TEWL and skin hydration are parameters that are often used to determine skin conditions and the barrier integrity of the skin. The data of this study showed that high TEWL and/or high skin hydration values alone are not sufficiently representative to securely identify barrier-impaired skin because rigor mortis causes high TEWL values and high skin hydration, but reduced skin permeability instead. Additional parameters are, therefore, needed to discriminate between suitable and non-suitable skin areas for the performance of skin-penetration studies on porcine ears. Besides the pH of porcine ear meat, the AF of the skin was determined as such a parameter. More research is now needed to define exact values for these parameters that can be used in the future to decide if an ear should be included or excluded from the intended skin-penetration experiments.

Considering the findings of this study, it was possible to compare the penetration efficacy of curcumin from four different innovative formulation principles. SmartFilms and NLC were identified as superior formulation strategies for effective dermal and transdermal delivery of curcumin.

## Figures and Tables

**Table 1:** Overview of the formulations tested, their compositions and physical-chemical properties during the test period of the study.

Formulation	Code	Composition	Physical- Chemical Properties	Reference
curcumin nanosuspension	Cur-NS	curcumin 5.0% D-α-tocopherol-polyethylenglycol-1000-succinate (TPGS) 1.0% water ad 100%	z-average: 260 nm d(v)0.9: 0.8 µm d(v)0.95: 1.4 µm ZP: −11 mV	[40]
curcumin nanosuspension in xanthan gel	Cur-Gel	curcumin 1.0% TPGS 0.2% xanthan gum 2.0% water ad 100%	d(n)0.9: 1.2 µm d(n)0.95: 1.6 µm viscosity: 0.9 Pas	[40]
DiI-NLC	DiI-NLC	DiI perchlorate * 0.005% sea fish oil 4.0% glyceryl tristearate 6.0% aqueous phase ** ad 100%	z-average: 165 nm d(v)0.9: 0.3 µm d(v)0.95: 0.4 µm ZP: −18 mV	[41]
curcumin-loaded smartFilms	smartFilms	curcumin with 152 µg curcumin/cm^2^ in cellulose matrix (paper)	curcumin in amorphous state in cellulose fibers	[42]
curcumin-NLC	Cur-NLC	curcumin 0.1%, medium-chain triglycerides 2.0% cetylpalmitate 8.0% alcyl polyglycoside 818 1.0%, water ad 100%	z-average: 163 nm d(v)0.9: 0.2 µm d(v)0.95: 0.3 µm ZP: −37 mV	[43]
6-carboxy- fluorescein in collagen gel	CF-Gel	6-carboxyfluorescein 0.1%, sponge collagen gel ad 100%	CF homogeneously dissolved in gel	[10]

* 1,1′-dioctadecyl-3,3,3′,3′-tetramethylindo-carbocyanin perchlorate. ** aqueous phase consisted of poloxamer 188 (3.0%), polysorbate 80 (0.1%), polyvinyl pyrrolidone 10,000 (0.1%), TPGS (1.0%) and purified water (ad 100%). ZP = zeta potential.

**Table 2:** Experimental design of the study.

Code	Post-Slaughter Age of Ears during Use *	Storage of Ear	Trial 1 Week 1	Trial 2 Week 2	Trial 3 Week 3	Trial 4
A	4–12 h	fresh ears used at day of slaughter	A-1	A-2	A-3	A-4
B	24–36 h	fresh ears stored for 24 h at 4 °C before use	B-1	B-2	B-3	
C	48–60 h	fresh ears stored for 48 h at 4 °C before use	C-1	C-2	C-3	
D	1–6 months	frozen ears (−20 °C), defrosted 12 h before use				D

* The dermal penetration efficacy was assessed for the six different formulations, and the TEWL, skin hydration, skin friction, AOC, AF of skin and SCT of untreated skin were determined.

**Table 3:** Application scheme for the different topical formulations.

Code	Dose Applied	Mode of Application	Ears
skin test A	-	area for determination of TEWL, hydration and skin friction	1–3
blank A	-	untreated skin	1–3
Cur-NS	50 µL	3-min finger massage	1–3
Cur-Gel	250 mg	3-min finger massage	1–3
DiI-NLC	50 µL	30-s finger massage	1–3
skin test B	-	area for determination of TEWL, hydration and skin friction	4–6
blank B	-	untreated skin	4–6
smartFilms	1.5 × 1.5 cm	fixation of wettened film to skin with breathable patch	4–6
Cur-NLC	10 µL	no massage	4–6
CF-Gel	50 mg	3-min finger massage	4–6
AOC	-	untreated skin as area for determination of AOC	4–6

**Table 4:** Microscopic settings for the different formulations used for image acquisition.

Code	Filter Block	Intensity of Light Source	Exposure Time
Cur-NS	excitation filter: 340–390 nm, dichroic mirror: 410 nm, emission filter: starting at 420 nm (LP)	50%	50 ms
Cur-Gel	excitation filter: 340–390 nm, dichroic mirror: 410 nm, emission filter: starting at 420 nm (LP)	50%	50 ms
DiI-NLC	excitation filter: 540–560 nm, dichroic mirror: 570 nm, emission filter: starting at 580 nm (LP)	100%	50 ms
smartFilms	excitation filter: 460–490 nm (BP), dichroic mirror: 500 nm, emission filter: starting at 500 nm (LP)	50%	50 ms
Cur-NLC	excitation filter: 460–490 nm (BP), dichroic mirror: 500 nm, emission filter: starting at 500 nm (LP)	50%	50 ms
CF-Gel	excitation filter: 460–490 nm (BP), dichroic mirror: 500 nm, emission filter: starting at 500 nm (LP)	25%	33 ms
blank A/B	all above	50%, 100%	50 ms

**Table 5:** Comparison of applied curcumin dose and dermal penetration efficacy of curcumin from different formulations.

Code	Amount Applied to Skin [µg]	MPD [µm]	TAP [MGV/Pixel]	MPD per µg Cur rel. to Cur-NS	TAP per µg Cur rel. to Cur-NS
Cur-NS	2500	16 ± 13	0.6 ± 0.2	1	1
Cur-Gel	2500	38 ± 30	0.6 ± 0.2	2	1
smartFilms	342	234 ± 52	15.6 ± 3.7	107	190
Cur-NLC	10	48 ± 24	2.4 ± 1.5	763	989

## Data Availability

Not applicable.

## Supplementary Materials

The following supporting information can be downloaded at: https://www.mdpi.com/article/10.3390/pharmaceutics14030678/s1, Section S1: Macro used for the determination of the AF-dermis, Section S2: Automated threshold algorithms, Section S3: Influence of porcine ear age on penetration efficacy for the six different formulations tested—detailed data, Section S4: Influence of porcine ear age on penetration efficacy for the Cur-Gel—selected data.

## Appendix A

List of Abbreviations

AF	autofluorescence
AF-dermis	autofluorescence of the dermis
AF-SC	autofluorescence of the stratum corneum
AF-total	autofluorescence of the whole skin cut (image)
AOC	antioxidant capacity
ATP	adenosine triphosphate
BP	band pass
CF	6-carboxyfluorescein
CF-Gel	6-carboxyfluorescein in collagen gel
Cur	curcumin
Cur-Gel	curcumin nanosuspension in xanthan gel
Cur-NLC	curcumin-NLC
Cur-NS	curcumin nanosuspension
DFD	dark, firm and dry
DIA	digital image analysis
DiI	1,1′-dioctadecyl-3,3,3′,3′-tetramethylindo-carbocyanin perchlorate
DiI-NLC	DiI-NLC
LP	long pass
MGV	mean gray value
MPD	mean penetration depth
NAD(P)H	nicotinamide adenine dinucleotide phosphate
NLC	nanostructured lipid carriers
ORAC	oxygen-radical absorbance-capacity assay
PBS	phosphate-buffered saline
PSE	pale, soft and exudative
SC	stratum corneum
SCT	stratum corneum thickness
SD	standard deviation
smartFilms	curcumin-loaded smartFilms
TAP	total amount penetrated
TE	Trolox equivalent
TEWL	transepidermal water loss
ZP	zeta potential
