# Supplementary material for: Assessing the Dermal Penetration Efficacy of Chemical Compounds with the Ex-Vivo Porcine Ear Model
Source: Pharmaceutics. 2022 Mar 19;14(3):678. doi: 10.3390/pharmaceutics14030678 (PMC8951478; doi:10.3390/pharmaceutics14030678)
Supplement: Supplementary file 1 [file pharmaceutics-14-00678-s001.zip › pharmaceutics-1632896-supplementary.pdf]

pharmaceutics

Supplementay Materials: Assessing the Dermal Penetration Efficacy of Chemical Compounds with the Ex-Vivo Porcine Ear Model

Cornelia M. Keck *, Ayat Abdelkader, Olga Pelikh, Sabrina Wiemann, Vasudha Kaushik, David Specht, Ralph W. Eckert, Reem M. Alnemari, Henriette Dietrich and Jana Brüßler

Supplementary material – Section S1

The macro used for the determination of the AF-dermis was:

makeRectangle(30, 1302, 1866, 80);
run("Crop");
run("Measure");

Supplementary material – Section S2

Table S1: All images were subjected to an automated threshold algorithms. Five different algorithms were programmed (A1-5) with ImageJ software. Each algorithm consisted of two macros that were run subsequently. A1 was used for Cur-NS and Cur-Gel, A2 for Dil-NLC, A3 for the smartFilms, A4 for the Cur-NLC and A5 for the CF-Gel (cf. Table 1 in the original manuscript).

A1	A2	A3	A4	A5
Macro 1				
// Color Thresholder 1.52a<br>// Autogenerated macro, single images only!<br>min=newArray(3);<br>max=newArray(3);<br>filter=newArray(3);<br>a=getTitle();<br>run("RGB Stack");<br>run("Convert Stack to Images");<br>selectWindow("Red");<br>rename("0");<br>selectWindow("Green");<br>rename("1");<br>selectWindow("Blue");<br>rename("2");<br>min[0]=33;<br>max[0]=255;<br>filter[0]="pass";<br>min[1]=0;	// Color Thresholder 1.53a<br>// Autogenerated macro, single images only!<br>min=newArray(3);<br>max=newArray(3);<br>filter=newArray(3);<br>a=getTitle();<br>run("RGB Stack");<br>run("Convert Stack to Images");<br>selectWindow("Red");<br>rename("0");<br>selectWindow("Green");<br>rename("1");<br>selectWindow("Blue");<br>rename("2");<br>min[0]=80;<br>max[0]=255;<br>filter[0]="pass";<br>min[1]=0;	// Color Thresholder 1.52a<br>// Autogenerated macro, single images only!<br>min=newArray(3);<br>max=newArray(3);<br>filter=newArray(3);<br>a=getTitle();<br>run("RGB Stack");<br>run("Convert Stack to Images");<br>selectWindow("Red");<br>rename("0");<br>selectWindow("Green");<br>rename("1");<br>selectWindow("Blue");<br>rename("2");<br>min[0]=0;<br>max[0]=0;<br>filter[0]="stop";<br>min[1]=33;	// Color Thresholder 1.53a<br>// Autogenerated macro, single images only!<br>min=newArray(3);<br>max=newArray(3);<br>filter=newArray(3);<br>a=getTitle();<br>run("RGB Stack");<br>run("Convert Stack to Images");<br>selectWindow("Red");<br>rename("0");<br>selectWindow("Green");<br>rename("1");<br>selectWindow("Blue");<br>rename("2");<br>min[0]=33;<br>max[0]=255;<br>filter[0]="pass";<br>min[1]=0;	// Color Thresholder 1.53a<br>// Autogenerated macro, single images only!<br>min=newArray(3);<br>max=newArray(3);<br>filter=newArray(3);<br>a=getTitle();<br>run("RGB Stack");<br>run("Convert Stack to Images");<br>selectWindow("Red");<br>rename("0");<br>selectWindow("Green");<br>rename("1");<br>selectWindow("Blue");<br>rename("2");<br>min[0]=0;<br>max[0]=0;<br>filter[0]="stop";<br>min[1]=60;

max[1]=0;<br>filter[1]="stop";<br>min[2]=0;<br>max[2]=0;<br>filter[2]="stop";<br>for (i=0;i<3;i++){<br>selectWindow(""+i);<br>setThreshold(min[i], max[i]);<br>run("Convert to Mask");<br>if (filter[i]=="stop") run("Invert");<br>}<br>imageCalculator("AND create", "0","1");<br>imageCalculator("AND create", "Result of 0","2");<br>for (i=0;i<3;i++){<br>selectWindow(""+i);<br>close();<br>}<br>selectWindow("Result of 0");<br>close();<br>selectWindow("Result of Result of 0");<br>rename(a);<br>// Colour Thresholding--------------	max[1]=255;<br>filter[1]="pass";<br>min[2]=0;<br>max[2]=0;<br>filter[2]="stop";<br>for (i=0;i<3;i++){<br>selectWindow(""+i);<br>setThreshold(min[i], max[i]);<br>run("Convert to Mask");<br>if (filter[i]=="stop") run("Invert");<br>}<br>imageCalculator("AND create", "0","1");<br>imageCalculator("AND create", "Result of 0","2");<br>for (i=0;i<3;i++){<br>selectWindow(""+i);<br>close();<br>}<br>selectWindow("Result of 0");<br>close();<br>selectWindow("Result of Result of 0");<br>rename(a);<br>// Colour Thresholding--------------	max[1]=255;<br>filter[1]="pass";<br>min[2]=0;<br>max[2]=0;<br>filter[2]="stop";<br>for (i=0;i<3;i++){<br>selectWindow(""+i);<br>setThreshold(min[i], max[i]);<br>run("Convert to Mask");<br>if (filter[i]=="stop") run("Invert");<br>}<br>imageCalculator("AND create", "0","1");<br>imageCalculator("AND create", "Result of 0","2");<br>for (i=0;i<3;i++){<br>selectWindow(""+i);<br>close();<br>}<br>selectWindow("Result of 0");<br>close();<br>selectWindow("Result of Result of 0");<br>rename(a);<br>// Colour Thresholding--------------	max[1]=255;<br>filter[1]="pass";<br>min[2]=0;<br>max[2]=0;<br>filter[2]="stop";<br>for (i=0;i<3;i++){<br>selectWindow(""+i);<br>setThreshold(min[i], max[i]);<br>run("Convert to Mask");<br>if (filter[i]=="stop") run("Invert");<br>}<br>imageCalculator("AND create", "0","1");<br>imageCalculator("AND create", "Result of 0","2");<br>for (i=0;i<3;i++){<br>selectWindow(""+i);<br>close();<br>}<br>selectWindow("Result of 0");<br>close();<br>selectWindow("Result of Result of 0");<br>rename(a);<br>// Colour Thresholding--------------	max[1]=255;<br>filter[1]="pass";<br>min[2]=0;<br>max[2]=0;<br>filter[2]="stop";<br>for (i=0;i<3;i++){<br>selectWindow(""+i);<br>setThreshold(min[i], max[i]);<br>run("Convert to Mask");<br>if (filter[i]=="stop") run("Invert");<br>}<br>imageCalculator("AND create", "0","1");<br>imageCalculator("AND create", "Result of 0","2");<br>for (i=0;i<3;i++){<br>selectWindow(""+i);<br>close();<br>}<br>selectWindow("Result of 0");<br>close();<br>selectWindow("Result of Result of 0");<br>rename(a);<br>// Colour Thresholding--------------
Macro 2				
run("Invert");	run("Invert");	run("Invert");	run("Invert");	run("Invert");

Supplementary material – Section S3

Table S2: Influence of porcine ear age on penetration efficacy for the six different formulations tested (cf. table 2). A= fresh ears, B = ears stored for 24-36h, C= ears stored for 48-60h, D = frozen and on demand defrosted ears. Data obtained from the different trials (week 1-3) are shown as mean ± SE in separate lines to visualize the variations in parameters caused by post-slaughter age of the ears (A-C) and by the different time points of testing (trials 1-3 = week 1-3).

	Cur-NS	Cur-Gel	Dil-NLC	smartFilms	Cur-NLC	CF-Gel
MPD [µm]	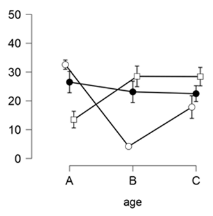	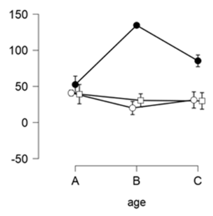	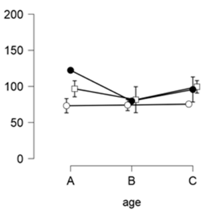	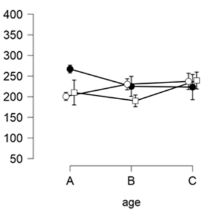	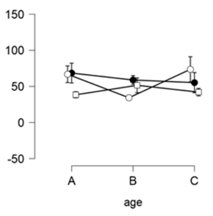	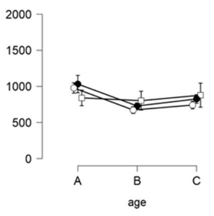
TAP [MGV/px]	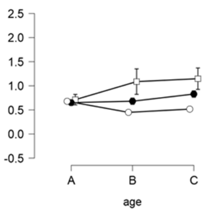	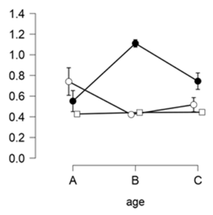	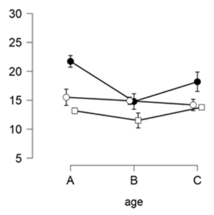	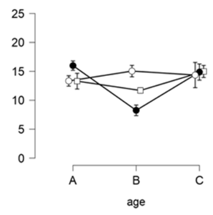	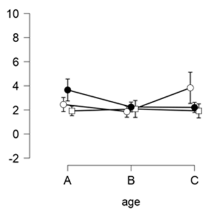	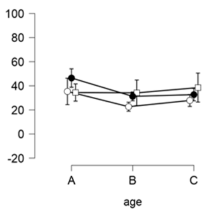

○ data trial 1 ● data trial 2 □ data trial 3

Supplementary material – Section S4

Table S3. Influence of porcine ear age on penetration efficacy for the Cur-Gel. A= fresh ears, B = ears stored for 24-36h, C= ears stored for 48-60h, D = frozen and on demand defrosted ears. A: original data from Fig. 4 comprising the MPD and TAP data for the Cur-Gel from all ears tested (33 biopsies treated with Cur-Gel in total). B: data after exclusion of data from two ears (2 from 33) that led to extremely high penetration values (cf. text). After elimination of the data, the trend is similar to the other formulations tested (the use of B-ears results in lower penetration efficacy).

	**A: Cur-Gel**<br>**original data**	**B: Cur-Gel**<br>**after exclusion of data from two ears with extremely high values**
**MPD [µm]**	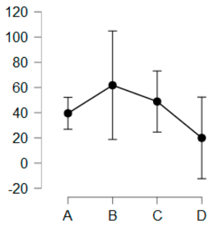	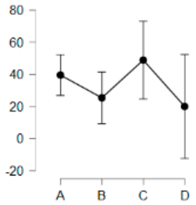
**TAP [MGV/pixel]**	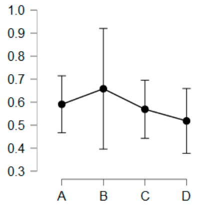	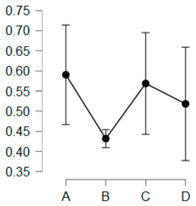
